# Fungistatic and Fungicidal Capacity of a Biosurfactant Extract Obtained from Corn Steep Water

**DOI:** 10.3390/foods9050662

**Published:** 2020-05-20

**Authors:** Alejandro López-Prieto, Xanel Vecino, Lorena Rodríguez-López, Ana Belén Moldes, José Manuel Cruz

**Affiliations:** 1 Chemical Engineering Department, School of Industrial Engineering–Industrial and Technology Research Centre (MTI), University of Vigo, Campus as Lagoas-Marcosende, 36310 Vigo, Spain; alexlopez@uvigo.es (A.L.-P.); lorena@uvigo.es (L.R.-L.); jmcruz@uvigo.es (J.M.C.); 2 Chemical Engineering Department, Polytechnic University of Catalunya (UPC)–Barcelona TECH, Barcelona Research Center for Multiscale Science and Engineering, Campus Diagonal–Besòs, 08930 Barcelona Spain; xanel.vecino@upc.edu

**Keywords:** corn stream, biosurfactant, fungicide, *Aspergillus brasiliensis*, *Candida albicans*

## Abstract

Biosurfactants are surface-active compounds that are produced by microorganisms, which in addition to their surfactant capacity, can possess interesting antimicrobial activities that are used in their incorporation into the agrifood industry. In this work, the preservative capacity of a novel biosurfactant extract obtained from a residual stream of the corn-milling industry was evaluated against two different fungi (*Aspergillus brasiliensis* and *Candida albicans*) under different biosurfactant concentrations (0.33–0.99 mg/mL), temperatures (4–40 °C), and incubation times (5–11 days). All the assays started with the same concentration of fungi (2 × 10^6^ CFU/mL). The results showed that temperature played an important role in the bactericidal and fungistatic effects of this biosurfactant extract. It was observed that at a low biosurfactant concentration (0.33 mg/mL) and low or high temperatures in the range tested, this biosurfactant extract possessed an important fungicidal effect (complete inhibition) on *A. brasiliensis*, while at intermediate temperatures, it achieved a fungistatic effect (50% of inhibition). Regarding *C. albicans*, it was observed that this strain was more resistant than *A. brasiliens*, although it was possible to achieve growth inhibitions of 76.3% at temperatures of 40 °C after 8 days of incubation with a biosurfactant concentration of 0.99 mg/mL. This work supports the possible application of biosurfactants extracted from corn steep water as preservatives and antimicrobial agents against fungal contaminations on agrifood products.

## 1. Introduction

It is estimated that microbial spoilage is responsible for the loss of almost 33% of the global annual food production [1], resulting in 1.3 billion tons of food waste worldwide [2]. Some of these microbial contaminations are produced by pathogenic fungal strains, among which, some of the most common are *Aspergillus* and *Candida* spp. These pathogens can be commonly found in soil, as well as in a wide range of crops, such as maize, lettuce, onion, tomato, and other vegetables [3,4]. Some *Aspergillus* spp., including *A. fumigatus*, *A. niger*, or *A. flavus*, which can behave as opportunistic pathogens, are known to affect animal and human health. They are responsible for causing food poisoning through the production of mycotoxins, as well as illnesses like aspergillosis, which is a pulmonary disease with symptoms of hemoptysis (coughing up blood) and chronic coughing [5,6].

On the other hand, *Candida albicans* is an opportunistic pathogen that is widely known for being the most pathogenic yeast species and responsible for causing infections in humans by colonizing oral cavities through saliva and oral mucosa, resulting in oropharyngeal candidiasis or oral thrush disease; *C. albicans* is especially virulent in immune-compromised patients [7]. Moreover, the pathogenicity of *C. albicans* can be related to its capacity for producing biofilms that act as protective structures for the microorganism [8]. Therefore, it can be speculated that if a fungicide is effective against *C. albicans*, it should also be effective against food spoilage by *Candida* species, which possess fewer resistant mechanisms. Fungal contaminations are related to the production of a wide range of crops, where pesticides are essential for avoiding the growth of these pathogens. In the last few years, consumer demands and European Union (EU) regulations have led the industry to search for more environmentally friendly pesticides. New pesticide formulations with biodegradable and eco-friendly compounds that are harmless to humans are needed to promote a healthy food chain. Amide and copper-based pesticides are found among the compounds that are banned by the European Commission (EC) [9].

Additionally, the increasing demand for the use of more biocompatible and biodegradable compounds in food formulations has led the food industry to research new additives from renewable and natural sources [10]. In this regard, biosurfactants, which are obtained by microbial production as secondary metabolites, many of them with antimicrobial activity, appear as an alternative for the food industry to surfactants and preservatives obtained via chemical synthesis. Indeed, biosurfactants show less toxicity and more efficiency than their chemical counterparts [11,12,13,14]. Due to their composition, constituted mainly by lipids, proteins, and/or sugars [11,12], in addition to their surfactant and antimicrobial activities, they have been studied and applied in the last decade in a wide range of applications, such as in the pharmaceutical, cosmetic, and food industries [14,15,16,17]. However, only a few publications have addressed the application of biosurfactants for food products. For instance, López-Prieto et al. [14] showed that a biosurfactant extract, obtained from an agro-industrial residue of the corn-milling industry, was able to promote the growth of *Lactobacillus casei* contained in a drinkable yogurt.

It is worth mentioning that biosurfactants have high antimicrobial and anti-adhesive activities against pathogenic strains. For example, biosurfactants produced by *Lactobacillus* spp.–such as *L. paracasei* [18], *L. helveticus* [19], or *L. pentosus* [20]–showed antimicrobial properties against foodborne pathogens–such as bacteria like *Pseudomonas aeruginosa*, *Staphylococcus aureus*, and *Escherichia coli*, and fungi like *A. brasiliensis* (also known as *A. niger*) and *C. albicans*. Additionally, López-Prieto et al. [21] showed that a biosurfactant extracted from a corn stream was effectively able to work as a bactericidal agent against pathogens, such as *P. aeruginosa* and *E. coli*, inhibiting their microbial growth at concentrations around 1 mg/mL. Moreover, the ability of biosurfactants in the removal of pathogenic biofilms from *E. coli* or *C. albicans* [22,23,24], and in the reduction of the bacterial pathogens’ adhesion to voice prostheses or silicone rubber [25], have also been reported.

On the other hand, agro-industrial residues represent an important source for the production of biosurfactants, providing and increasing their added value as secondary raw materials. For instance, corn steep water (CSW), a fermented aqueous stream from the corn-milling industry, can be a source of biosurfactants. Biosurfactant extracts from CSW are produced via spontaneous fermentation due to the steeping process of corn in the presence of SO_2_ under acidic conditions and between temperatures of 45 °C and 52 °C [26]. Recently, it has been demonstrated that *Bacillus* strains are responsible for the production of biosurfactants in CSW under these extreme conditions [27]. Extracellular biosurfactants from CSW can be extracted using liquid-liquid (L-L) extraction with ethyl acetate or chloroform [28]. Although the extraction with chloroform was shown in some publications, the EU regulation allows the use of ethyl acetate, but not chloroform, in L-L extractions for food applications [29].

Regarding the properties of biosurfactants extracted from CSW, it has been proven that biosurfactant extracts from CSW have multifunctional properties, such as surfactant and antioxidant capacities [16], as well as bactericidal activity [22]. Furthermore, they were able to reduce the surface tension (mN/m) of water to around 30 units [29], and were identified as lipopeptides with a critical micellar concentration (CMC) between 100–400 mg/L [22] and a composition of C16 and C18 fatty acids [16].

Therefore, this work aimed to evaluate the fungistatic and fungicidal activity of an extracellular biosurfactant extract, obtained using liquid-liquid extraction with ethyl acetate from CSW, on foodborne pathogens (*A. brasiliensis* and *C. albicans*).

## 2. Materials and Methods

### 2.1. Extraction of Biosurfactants from Corn Steep Water

Liquid-liquid extraction with organic solvents was carried out following the methodology described by Vecino et al. [28] to obtain an extracellular biosurfactant from CSW, which was provided by FeedStimulants (Reg. No. NL214247, Lot NL-2728DK 7). Briefly, the biosurfactant was extracted with ethyl acetate from CSW (CSW solution: ethyl acetate 1:3 (v/v)) at room temperature (25 ± 1 °C) for 60 min. Ethyl acetate was supplied by CARLO ERBA Reagents, S.A.S (Val de Reuil Cedex, France). Then, the organic solvent was evaporated using vacuum distillation and recycled for further extractions, obtaining a multifunctional biosurfactant extract that was dissolved in distilled water at different concentrations (0.33–0.99 mg/mL). Afterward, a 0.22 μm Stericup^®^ 150 mL Durapore^®^ polyvinylidene fluoride (PVDF) membrane (EMD Millipore Corporation, Billerica, MA, USA) was used to filter the biosurfactant extract to evaluate its antifungal capacity against *A. brasiliensis* and *C. albicans*. All steps of the process were conducted under sterile conditions. The extractive yield for the extracellular biosurfactant extraction from the CSW was determined gravimetrically by weighing a sample before and after drying at 100 °C for 48 h in a stove following the methodology described by Rodríguez-López et al. [12].

### 2.2. Surface Activity and Critical Micellar Concentration Determination–Wilhelmy Plate Assay

The determination of the surface tension (ST) and CMC of the extracellular biosurfactant extracted with ethyl acetate from the CSW was achieved by using a Krüss K20 EasyDyne tensiometer with a 1.9 cm platinum Wilhelmy plate (Krüss GmbH, Hamburg, Germany). They were prepared using several dilutions in distilled water to determine the CMC of the biosurfactant extract from the CSW. All measurements were carried out in triplicate at room temperature and all the results are shown as the mean value and standard deviation of all three measurements.

### 2.3. Elemental Analysis of the Extracellular Biosurfactant Obtained from the Corn Steep Water

Elemental analysis was performed to determine the C, H, N, and S composition of the biosurfactant extract obtained from the CSW using an elemental analyzer (Fisons Carlo Erba EA-1108 CHNS-O, LabX, Midland, ON, Canada). To obtain the amount of N, a correlation with the protein content was conducted by multiplying it by a factor of 6.25, based on a previous study [30].

### 2.4. Strains and Standard Culture Conditions for the Antimicrobial Assay

The antimicrobial activity of the extracellular biosurfactant obtained from the CSW was assessed against two pathogenic fungal strains obtained from the Spanish Type Culture Collection (CECT) (Valencia, Spain). The strains selected were the following: *Aspergillus brasiliensis* CECT-2574 (ATCC-16404) and *Candida albicans* CECT-1392 (ATCC-2091). Both strains were cultivated in potato dextrose broth (PDB) medium at 22 °C for 5 days in aerobic conditions in 250 mL Erlenmeyer flasks at 150 rpm. The composition of the PDB medium was 4 g/L potato peptone and 20 g/L of glucose.

### 2.5. Antimicrobial Assay

The antimicrobial activity of the biosurfactant extracted with ethyl acetate from the CSW against two pathogenic fungal strains of *A. brasiliensis* and *C. albicans* was determined by measuring the optical density at 600 nm in 96-well plates in a microplate reader (MultiSkan GO Microplate Photometer, ThermoFisher Scientific, Waltham, MA, USA) following the methodology described by Vecino et al. [20].

Briefly, PDB medium containing different concentrations of the biosurfactant extract from the CSW (0.33–0.99 mg/mL) were prepared in 10 mL sterile tubes with a final sample volume of 2 mL. An inoculum of 20 µL of the selected fungal pathogen, with a final concentration of microorganisms of 2 × 10^6^ colony-forming units (CFU)/mL, was used for each of the experiments. All samples were prepared in triplicate. Likewise, positive controls consisted of PDB medium containing the fungal strain, whereas the negative control was formulated with only PDB medium (in the absence of the biosurfactant extracted from the CSW and the pathogenic strain). Every tube was then rinsed and incubated at three different temperatures of 4, 22, and 40 °C. After 5, 8, and 11 days, a volume of 250 µL of each of the samples and controls was placed into the columns of 96-well microplates and then the optical density was measured at 600 nm.

Percentages of growth inhibition of the fungal strains at different concentrations of the biosurfactant extracted from the CSW were calculated using Equation (1):

(1) % Growth Inhibition=[1−(AcA0)]×100

where *A_c_* represents the absorbance of the samples at the different concentrations of the biosurfactant extract from the CSW and *A*_0_ represents the absorbance of the positive control well in the absence of the biosurfactant extract. All the results are shown as the mean value and standard deviation of all three measurements of the triplicate.

### 2.6. Experimental Design

An incomplete Box-Behnken factorial design [31] was carried out to assess the antifungal activity of a biosurfactant extracted with ethyl acetate from the CSW against *A. brasiliensis* and *C. albicans* under different conditions of biosurfactant concentration, temperature, and incubation time.

Independent and dependent variables assessed in this work, along with their ranges established in the experiments, are shown in Table 1. The coded dimensionless independent variables used were defined as *x*_1_ (biosurfactant concentration), *x*_2_ (temperature), and *x*_3_ (time) with limits of variation between −1 and 1. The dependent variables were defined as *y*_1_ (% of growth inhibition of *A. brasiliensis*) and *y*_2_ (% of growth inhibition of *C. albicans*). To determine the relationship between the independent and dependent coded variables of this study within the ranges of variation established in Table 1, linear equations were applied (Equation (2)):

(2) $$x_{i}=\left(\frac{z_{i}-z_{i}^{0}}{\Delta z_{i}}\right)\beta_{d}$$

where Δ*z_i_* represents the distance between the real value obtained at the central point and the real value obtained at the upper or lower level of each variable, *β_d_* is the major coded limit value in the matrix for each variable, and *z_i_*^0^ is the real value in the central point.

Values of −1, 0, and +1 were assigned to each coded variable, which represent the minimum, central, and maximum values, respectively, for each variable of the experiment within the ranges established in Table 1.

The fungistatic and fungicidal capacity of the biosurfactant extracted from the CSW was determined for each of the two pathogenic strains as the lowest concentration of the biosurfactant that inhibited 50% of the fungal growth and produced complete inhibition (100%), respectively.

### 2.7. Statistical Analysis

The experimental data obtained in this study were analyzed using the response surface method with Design-Expert^®^ Version 12 (Stat-Ease, Inc., Minneapolis, MN, USA) by fitting the results obtained to a quadratic function shown in Equation (3):

(3) $$y=\beta_{0}+\beta_{1}x_{1}+\beta_{2}x_{2}+\beta_{3}x_{3}+\beta_{12}x_{1}x_{2}+\beta_{13}x_{1}x_{3}+\beta_{23}x_{2}x_{3}+\beta_{11}x_{1}^{2}+\beta_{22}x_{2}^{2}+\beta_{33}x_{3}^{2}$$

where *y* is the dependent variable of each experiment (% of growth inhibition of *A. brasiliensis* or *C. albicans*, respectively); *β_i_* represents the regression coefficients, which were calculated from experimental data by performing multiple regressions using the least-squares method; and *x_i_* represents the independent variables of this study (biosurfactant concentration, temperature, and time of incubation).

This equation predicts the percentage of growth inhibition of *A. brasiliensis* and *C. albicans* within the range of the independent variables considered in the Box-Behnken factorial design shown in Table 1. For the experiment conducted in this work, unlike a linear equation, a quadratic equation produces a parabola that begins at a single point, called the vertex, and extends upward or downward in the y-direction. In this particular design, there is no one-to-one relationship between *x* and *y*, because it results in two values of *x* for any given value of *y*, except for the *y* value corresponding to the vertex point; this is in contrast with a linear equation, where this relationship is one-to-one since each *x* value produces only one *y* value [31].

## 3. Results and Discussion

### 3.1. Biosurfactant Characterization

Regarding its physico-chemical characteristics, the biosurfactant extract from the CSW revealed its ability to reduce the surface tension of water to a minimum value of 43.8 mN/m, which is in concordance with the results shown in previous studies [12,15,21]. Additionally, the CMC obtained for the biosurfactant extract was 306.5 mg/L. Regarding the elemental analysis, the N, C, H, and S content showed that C was the element present in the highest proportion with 43.67%, while the contents of N, H, and S were 0.77%, 5.89%, and less than 0.30%, respectively, all of which are in the range of values of previous analysis performed on biosurfactants from CSW [21].

### 3.2. Antimicrobial Activity

The antimicrobial activity of the biosurfactant extracted with ethyl acetate from the CSW was determined by carrying out an incomplete Box-Behnken factorial design to assess the effect of different conditions (biosurfactant concentration *x*_1_, temperature *x*_2_, and time *x*_3_) to measure the growth inhibition percentages against two pathogenic fungal strains of *A. brasiliensis* and *C. albicans*. The experiments were conducted under static conditions to simulate real situations and to test the reproducibility of the study in a real setting. The biosurfactant concentrations used in the factorial design were chosen based on previous experiments of the evaluation of the bactericidal effect of a biosurfactant extract from CSW [21]. The range of temperatures was selected to be between refrigeration storage (4 °C) and a maximum temperature outside on a hot day (40 °C). The time for incubation was chosen based on the minimum days of incubation needed for fungal strains to grow, which was 5 days, up to a maximum of 11 days. The dependent variables evaluated were the growth inhibition percentages (*y*_1_, *y*_2_) of *A. brasiliensis* and *C. albicans*, as shown in Table 1. The experimental data obtained for the growth inhibition of *A. brasiliensis* (*y*_1_) and *C. albicans* (*y*_2_) in the 15 experiments carried out in triplicate at different concentrations of biosurfactant (*x*_1_), temperature (*x*_2_), and time of incubation (*x*_3_) are exhibited in Table 2. The initial concentrations of *A. brasiliensis* and *C. albicans* were 2.0 × 10^6^ CFU/mL.

To reduce the possible effect of systematic errors associated with the experimental data obtained in the present work, a random testing sequence of the 15 experiments was carried out. Regression coefficients were calculated for experiments 1 to 12, whereas for experiments 13 to 15, the evaluated influence of experimental errors represented the central points of the independent variables within the range established in Table 1.

On the other hand, the significance of each coefficient was established using *p*-values for the variables *y*_1_ and *y*_2_ displayed in Table 3. Using these regression coefficients, equations were created to determine the values of the dependent variables studied within the ranges established before the experiments were undertaken (see Table 1). By replacing the significant coefficients in Equation (3), it was possible to obtain the values of each dependent variable under the different conditions of biosurfactant concentration (*x*_1_), temperature (*x*_2_), and time (*x*_3_) established previously in the Box-Behnken factorial design.

In the case of *A. brasiliensis*, the concentration of the biosurfactant (*x*_1_) and temperature (*x*_2_) were the most statistically significant variables (*p* < 0.05) influencing the growth inhibition of the fungal strain (see Table 3). Furthermore, the interaction of these two independent variables produced higher inhibition percentages against *A. brasiliensis*.

**Figure 1:**
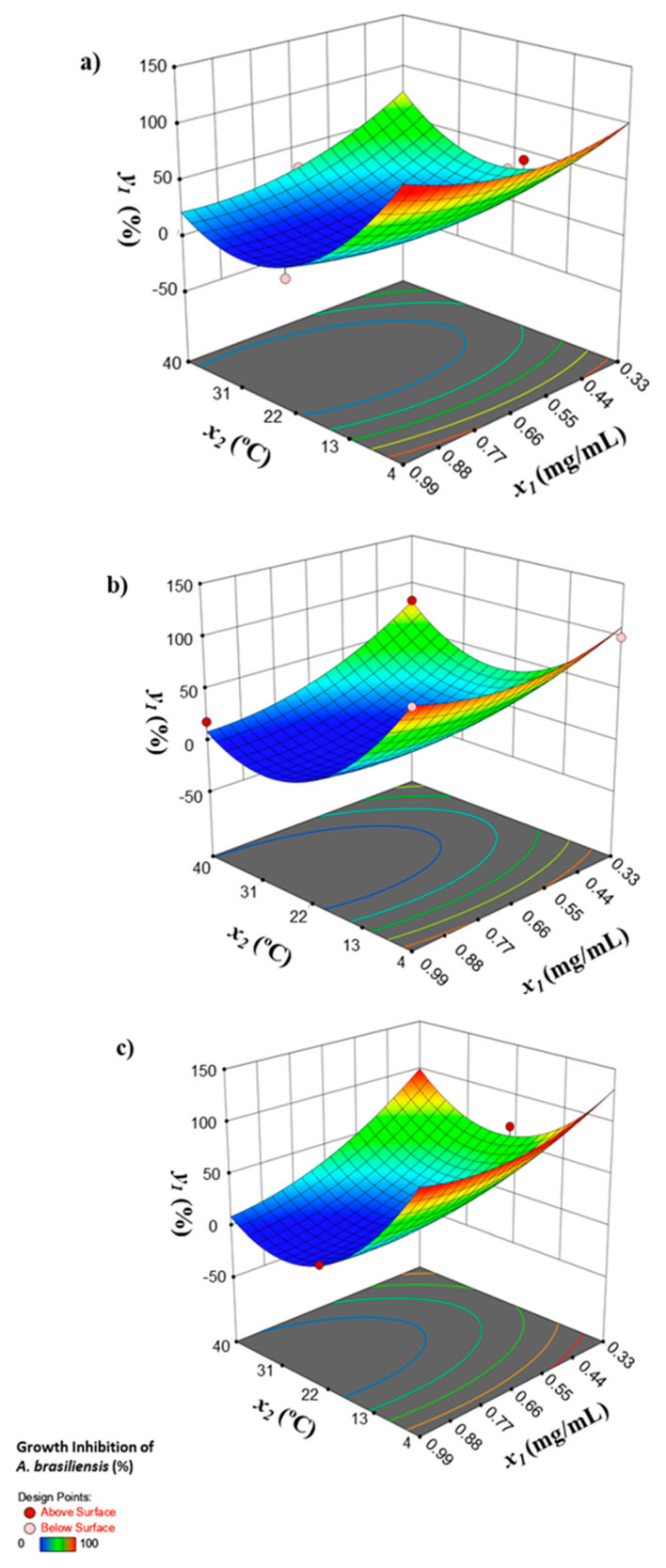
Growth inhibition of *A. brasiliensis* (%) as a function of the concentration of the biosurfactant (*x*_1_) and temperature (*x*_2_) of incubation for different incubation times (*x*_3_): (**a**) 5, (**b**) 8, and (**c**) 11 days.

Figure 1 shows the variation of the growth inhibition percentage of *A. brasiliensis* for the above-mentioned independent variables as the most statistically significant, namely the concentration of the biosurfactant (*x*_1_) and temperature (*x*_2_), with the time of incubation (*x*_3_) fixed at 5 (Figure 1a), 8 (Figure 1b), and 11 days (Figure 1c). Among them, the temperature had a more significant effect on the growth inhibition of *A. brasiliensis*; therefore, when the incubation was carried out at a low temperature (4 °C) with a low biosurfactant concentration (0.33 mg/mL) for the range of storage time evaluated in this work, a complete inhibition (100%) of *A. brasiliensis* was achieved, as shown in Figure 1. These results showed the efficiency of the biosurfactant extracted from the CSW against *A. brasiliensis*, mainly at low temperatures; hence, the biosurfactant extract could be applied for the preservation of food products stored in refrigeration conditions. Additionally, at high temperatures (40 °C), a fungicidal effect (82.5% of growth inhibition) was achieved at a biosurfactant concentration of 0.33 mg/L. The factorial design also determined the optimal conditions established by the factorial design for both pathogenic strains at room temperature (25 °C), showing that a biosurfactant concentration of 0.99 mg/mL resulted in a maximum inhibition of 57.1% against *A. brasiliensis* after 11 days of incubation, achieving a fungistatic effect. These results agree with the results obtained by Rodríguez-López et al. [32], where a biosurfactant extracted from CSW with chloroform displayed a fungistatic effect on *A. brasiliensis* at a temperature of 22 °C.

Regarding *C. albicans*, all three independent variables–biosurfactant concentration (*x*_1_), temperature (*x*_2_), and time (*x*_3_)–were found to be statistically significant (*p* < 0.05), influencing the growth inhibition of the pathogenic strain (see Table 3). Additionally, the interaction between the biosurfactant concentration (*x*_1_) and incubation temperature (*x*_2_) was found to be statistically significant. The maximum growth inhibition achieved from the Box-Behnken factorial design was 76.34%, achieving a fungistatic effect at a biosurfactant concentration of 0.99 mg/mL and a temperature of 40 °C after 8 days of incubation, as shown in Table 2. At room temperature (25 °C), the results showed that the highest inhibition percentage of the fungal strain was 20.0% at a biosurfactant concentration of 0.99 mg/mL after 5 days of incubation. However, at a temperature of 4 °C, the maximum growth inhibition achieved against *C. albicans* was 17.79%, as shown in Table 2. These results agree with a previous study conducted by Rodríguez-López et al. [32], where the biosurfactant extract obtained from CSW after extraction with chloroform did not achieve a fungistatic effect on *C. albicans* at a temperature of 22 °C.

Based on the results obtained in this work, it can be established that the biosurfactant extracted from the CSW with ethyl acetate was much more effective against *A. brasiliensis* than *C. albicans*, showing promising results as an antifungal agent against the *Aspergillus* family. It can be speculated that low temperatures and the presence of the biosurfactant produced a synergic effect on the inhibition of *A. brasiliensis*, while in the case of *C. albicans*, this effect was observed at high temperatures. This might be because the optimal temperature for the growth of *C. albicans* (33–38 °C) [33] is slightly higher than the optimal temperature for the growth of *A. brasiliensis* (24–35 °C) [34].

It is well known that *Candida* spp. can produce biofilm layers that act as a protective structure for the microorganism [8], making them more resistant than other microorganisms. In this work, the biofilm formation was not investigated, although it can be speculated that if the biosurfactant extract from CSW could inhibit the growth of *C. albicans*, it would also inhibit the formation of biofilms.

Table 4 shows the operational conditions within the parameters established in the Box-Behnken factorial design that achieved fungistatic (50% of growth inhibition) and fungicidal (100% complete inhibition) effects of the biosurfactant extracted from the CSW on *A. brasiliensis* and *C. albicans* (at 4 and 25 °C). For *A. brasiliensis*, concentrations of 0.33 and 0.99 mg/mL of the biosurfactant extract resulted in inhibitions of 50% and 100% at 4 °C during an incubation period of 5 and 10 days, respectively, achieving fungistatic and fungicidal effects on the pathogen depending on the concentration of the biosurfactant extract and the incubation time. Similarly, when the concentration of the biosurfactant extract was increased to 0.99 mg/mL, a 100% inhibition effect on *A. brasiliensis* was achieved after 10 days of incubation at room temperature (25 °C). Additionally, the results obtained for the optimal conditions to achieve both fungistatic and fungicidal effects on *A. brasiliensis* within the range of temperatures of the Box-Behnken factorial design were temperatures of 15.5 °C for the 50% growth inhibition and 39.6 °C for complete inhibition using a biosurfactant concentration between 0.97–0.99 mg/mL during incubations of 8.4 and 10.9 days, respectively. On the other hand, for *C. albicans*, neither fungistatic nor fungicidal effects were achieved at 4 and 25 °C, where the maximum percentages of growth inhibition were 17.9% at 4 °C and 20.0% at 25 °C for the range of biosurfactant concentrations tested, as shown in Table 4. However, it was possible to inhibit 50% of the growth of *C. albicans* at 36.6 °C with a biosurfactant extract concentration of 0.97 mg/mL after 10.8 days of incubation, showing a fungistatic effect under these conditions.

**Figure 2:**
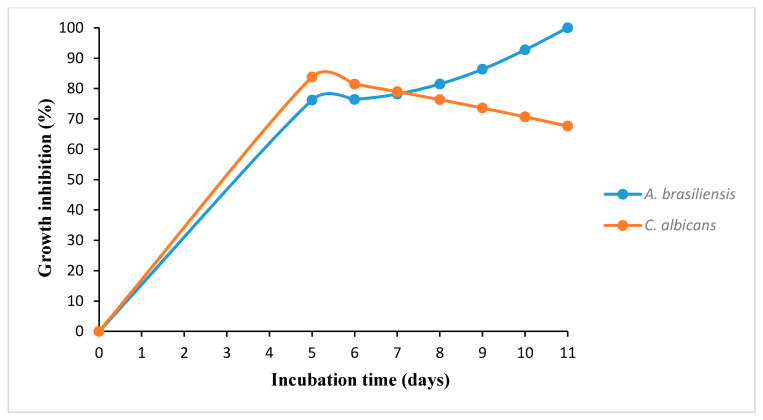
Growth inhibition kinetics of *A. brasiliensis* and *C. albicans* at a biosurfactant concentration of 0.99 mg/mL and temperature of 40 °C.

Additionally, the growth inhibition kinetics of *A. brasiliensis* and *C. albicans* were evaluated at the maximum biosurfactant concentration tested (0.99 mg/mL) at 40 °C over the incubation time tested (5–11 days), as shown in Figure 2. To obtain these results, the theoretical equation obtained in the factorial design was used. The r^2^ values obtained for the factorial design were 0.976 and 0.966 for *A. brasiliensis* and *C. albicans*, respectively. As can be observed in Figure 2, within the experimental period, the growth inhibition of *A. brasiliensis* was increased until it achieved complete inhibition, whereas *C. albicans* showed a higher resistance with a decrease in the growth inhibition during the period of incubation.

For comparative purposes, Table 5 summarizes those results obtained in previous works against *A. brasiliensis* and *C. albicans*. Only a few antimicrobial studies have carried out experiments with biosurfactants on *A. brasiliensis*. Table 5 also includes those microorganisms that can produce biosurfactants with antimicrobial activities against both *A. brasiliensis* and *C. albicans*. In most cases, high concentrations of biosurfactants were required to inhibit at least 50% of the growth of the pathogens, except for the study by Rodríguez-López et al. [32], who obtained almost complete inhibition of the growth of *A. brasiliensis* with concentrations of 1 mg/mL of a biosurfactant extracted from CSW with chloroform after 7 days of incubation. In contrast, for *C. albicans*, the same authors did not observe any growth inhibition during the whole experiment, which agrees with the results obtained in this work with the biosurfactant extracted from the CSW with ethyl acetate, where a different effect on *C. albicans* and *A. brasiliensis* was observed. Some authors have observed that cationic surfactants induce changes in the ionic character of the cell surface membrane from negative to positive, producing an antifungal effect [35]. The antimicrobial activity of the biosurfactant extract from the CSW evaluated in this work can be explained in terms of its amphoteric character, which has been proved in a previous study showing anionic and cationic charges [12].

Basit et al. [36] showed that a biosurfactant from a *Bacillus cereus* strain showed a fungistatic effect on both fungal pathogens at a concentration of 7 mg/mL, which is higher than the concentrations of the biosurfactant extract obtained from the CSW used in this work to obtain a fungistatic effect. Similar results were achieved for biosurfactants extracted with phosphate buffer saline (PBS) and phosphate buffer (PB) from *L. pentosus* and *L. paracasei* on *C. albicans* [18].

It is interesting to remark that even though the biosurfactant evaluated in this work did not achieve a fungicidal effect on *C. albicans*, it showed a higher efficiency in terms of the growth inhibition at lower concentrations than other biosurfactant extracts, such as those produced, for instance, from *Rhodococcus fascians* BD8 [37] (see Table 5). Moreover, in this work, at temperatures higher than 35 °C, it was possible to achieve a fungistatic effect. Additionally, Gudiña et al. [18] produced growth inhibitions over 50% on *C. albicans* with biosurfactants extracted from *L. paracasei* ssp. *paracasei* A20, although at higher concentrations than the ones used in this work (3.12–6.25 mg/mL). Moreover, biosurfactants from *L. helveticus* showed good fungistatic activity on *C. albicans*, although at concentrations around 25 mg/mL [19].

## 4. Conclusions

This is the first study concerning the possible application of a biosurfactant extracted from corn steep water with ethyl acetate as fungicidal and fungistatic agents against foodborne pathogens. The results obtained in this work showed the preservative and antimicrobial effects of a biosurfactant extract on pathogenic fungi like *A. brasiliensis* and *C. albicans*, which can be found on a wide range of crops and food products, resulting in massive losses due to their microbial contamination of agrifood products. Therefore, the biosurfactant extract could be considered as an additive with multifunctional properties for its application in the formulations of food products and crops. From the two experimental designs carried out in this study, it can be concluded that the biosurfactant extract was more effective against *A. brasiliensis* than *C. albicans*, especially at low storage temperatures. Finally, this work takes advantage of a circular economy since the biosurfactant extract was obtained from a secondary raw material of the agrifood industry with an important impact on the production of more biodegradable and sustainable preservatives and pesticides that would help to avoid food poisoning and contaminations.

## Figures and Tables

**Table 1:** Independent and dependent variables used in the study.

Variable	Nomenclature	Units	Range of Variation
(a) Independent variables			
Biosurfactant concentration	BS	mg/mL	0.33–0.99
Temperature	T	°C	4–40
Incubation time	t	days	5–11
**Variable**	**Nomenclature**	**Definition**	**Range of Variation**
(b) Dimensionless, coded independent variables			
Dimensionless BS	*x*_1_	(BS − 0.66)/0.33	(−1,1)
Dimensionless T	*x*_2_	(T − 22)/18	(−1,1)
Dimensionless t	*x*_3_	(t − 8)/3	(−1,1)
**Variable**		**Nomenclature**	**Units**
(c) Dependent variables studied			
Growth inhibition of *A. brasiliensis*		*y*_1_	%
Growth inhibition of *C. albicans*		*y*_2_	%

**Table 2:** Operational conditions used in this study, expressed as coded dimensionless and uncoded independent variables: concentration of the biosurfactant (*x*_1_), temperature (*x*_2_), and incubation time (*x*_3_); and the results obtained for the dependent variables: *y*_1_ (% of growth inhibition of *A. brasiliensis*) and *y*_2_ (% of growth inhibition of *C. albicans*). (−1, minimum value of the variable within the range; 0, central value of the variable within the range; 1, maximum value of the variable within the range).

	Coded Independent Variable			Uncoded Independent Variable			Dependent Variable	
Exp.	*x*_1_	*x*_2_	*x*_3_	*x*_1_ (mg/mL)	*x*_2_ (°C)	*x*_3_ (Days)	*y*_1_	*y*_2_
1	0	−1	−1	0.66	4	5	100.00	0.00
2	0	1	−1	0.66	40	5	31.71	62.55
3	0	−1	1	0.66	4	11	100.00	0.00
4	0	1	1	0.66	40	11	26.91	49.95
5	−1	−1	0	0.33	4	8	100.00	17.79
6	−1	1	0	0.33	40	8	82.52	40.89
7	1	−1	0	0.99	4	8	100.00	0.00
8	1	1	0	0.99	40	8	18.88	76.33
9	−1	0	−1	0.33	22	5	30.43	6.42
10	−1	0	1	0.33	22	11	67.87	0.00
11	1	0	−1	0.99	22	5	0.00	11.07
12	1	0	1	0.99	22	11	0.00	0.00
13	0	0	0	0.66	22	8	0.00	0.00
14	0	0	0	0.66	22	8	0.00	0.00
15	0	0	0	0.66	22	8	0.00	0.00

**Table 3:** Regression coefficients and their statistical significance for variables *y*_1_ (% of growth inhibition of *A. brasiliensis*) and *y*_2_ (% of growth inhibition of *C. albicans*). (*β*: regression coefficients of the linear, quadratic, and interactive effects of the independent variables studied).

	*y*_1_	*p_y_*_1_	*y*_2_	*p_y_*_2_
*β*_0_	0	0.0015 ^a^	0	<0.0001 ^a^
*β*_1_	20.24	0.0034 ^a^	2.79	0.0473 ^a^
*β*_11_	17.64	0.0272 ^a^	5.00	0.0243 ^a^
*β*_2_	−30	0.0006 ^a^	26.49	<0.0001 ^a^
*β*_22_	57.71	0.0002 ^a^	28.75	<0.0001 ^a^
*β*_3_	4.08	0.3411	−3.76	0.0168 ^a^
*β*_33_	6.94	0.2785	−0.6284	0.7053
*β*_12_	15.91	0.0338 ^a^	13.31	0.0003 ^a^
*β*_13_	9.36	0.1488	−1.16	0.4747
*β*_23_	−1.2	0.8355	−3.15	0.0909

^a^ Significant coefficient (*p* < 0.05).

**Table 4:** Fungicidal and fungistatic conditions of the biosurfactant extracted from the CSW against *A. brasiliensis* and *C. albicans* in refrigerator storage (4 °C) and room temperature (25 °C) (* fungistatic effect, ** fungicidal effect).

	*A. brasiliensis*			*C. albicans*			
T (°C)	t (Days)	Biosurfactant Concentration (mg/mL)	Growth Inhibition (%)	T (°C)	t (Days)	Biosurfactant Concentration (mg/mL)	Growth Inhibition (%)
4	5.0	0.33	50 *	4	5.0	0.99	17.9
	10.0	0.35	100 **				
25	10.2	0.99	50 *	25	5.0	0.99	20.0
	11.0	0.99	100 **				

**Table 5:** Comparison of antifungal activity of different biosurfactants from the literature against *A. brasiliensis* (also named *A. niger*) and *C. albicans*.

Microorganism.	Biosurfactant Type	Pathogenic Strain	Growth Inhibition (%)	Biosurfactant Concentration (mg/mL)	Extraction Method	Reference
*Bacillus*	Extracellular	*A. brasiliensis*	95	1	L-L extraction	Rodríguez-López et al. [32]
		*C. albicans*	0	1		
*B. cereus*	Not defined	*A. niger*	>50	7.6	MSM	Basit et al. [36]
		*C. albicans*	>50	7.6		
*L. paracasei* A20	Cell-bound	*C. albicans*	56.3	3.12	PBS	Gudiña et al. [18]
			65.3	6.25		
*Rhodococcus fascians* BD8	Extracellular	*C. albicans*	30	0.5	L-L extraction	Janek et al. [37]
			27	0.25		
		*C. albicans*	7	0.5		
			7	0.25		
*L. helveticus*	Cell-bound	*C. albicans*	<50	25	PBS	Sharma et al. [19]
*L. pentosus*	Cell-bound	*C. albicans*	<20	3.13	PBS	Vecino et al. [20]
	Cell-bound	*C. albicans*	<10	3.13	PB	
*L. paracasei*	Cell-bound	*C. albicans*	<5	3.13	PBS	
	Cell-bound	*C. albicans*	<20	3.13	PB	

Note: L-L—Liquid-liquid, MSM—Mineral salt medium, PB—Phosphate buffer, PBS—Phosphate buffer saline.
