# Correction: López-Prieto et al. Fungistatic and Fungicidal Capacity of a Biosurfactant Extract Obtained from Corn Steep Water. *Foods* 2020, *9*, 662

**DOI:** 10.3390/foods10061318

**Published:** 2021-06-08

**Authors:** Alejandro López-Prieto, Xanel Vecino, Lorena Rodríguez-López, Ana Belén Moldes, José Manuel Cruz

**Affiliations:** 1 Chemical Engineering Department, School of Industrial Engineering–Industrial and Technology Research Centre (MTI), University of Vigo, Campus as Lagoas-Marcosende, 36310 Vigo, Spain; alexlopez@uvigo.es (A.L.-P.); lorena@uvigo.es (L.R.-L.); jmcruz@uvigo.es (J.M.C.); 2 Chemical Engineering Department, Polytechnic University of Catalunya (UPC)–Barcelona TECH, Barcelona Research Center for Multiscale Science and Engineering, Campus Diagonal–Besòs, 08930 Barcelona, Spain; xanel.vecino@upc.edu

The authors would like to make the following correction to the published paper [1]: a few words should be corrected in the abstract, pages 7 and 9:

⚬ In the Abstract, “bactericidal” should be “fungicidal”.
⚬ In the Abstract, “low or high temperatures” should be “low temperatures”.
⚬ In the Abstract, “(50% of inhibition)” should be “(50% of inhibition) at the highest concentration”.
⚬ On page 7, in the sentence, “a fungicidal effect (82.5% of growth inhibition) was achieved at a biosurfactant concentration of 0.33 mg/mL”, “0.33 mg/mL” should be replaced by “0.99 mg/mL”.
⚬ On Page 9, “resulted in inhibitions of 50% and 100% at 4 °C during an incubation period of 5 and 10 days” should be “resulted in inhibitions of 100% and 57% at 4 °C and 25 °C, during an incubation period of 5 and 11 days”.
⚬ On Page 9, “a 100% inhibition effect on *A brasiliensis* was achieved after 10 days” should be “a 50% inhibition effect on *A. brasiliensis* was achieved after 10 days”.
⚬ On Page 9, “39.6 °C” should be “40 °C”.
⚬ On Page 9, “8.4 °C” should be “8.5 °C”.

Figure 1 and Table 2 and Table 4 in the published paper are not correct. Please see the correct versions as shown below.

The authors would like include a clarification in the manuscript regarding the fungicidal capacity of the biosurfactant extract under evaluation at 0.33 mg/mL. This clarification is not the result of an error on the part of the journal:

A short explanation should be included in the Results and Discussion section after the discussion of Table 4 regarding the fungicidal capacity of *A. brasiliensis*:

⚬ “It was observed that the biosurfactant extract under evaluation showed a fungicidal capacity at low concentrations of biosurfactant, 0.33 mg/mL, at 4 °C against *A. brasiliensis*. Therefore, it could be stated that this biosurfactant extract possesses good fungicidal properties to be used as a preservative in foods storage under refrigeration conditions”.

The Foods Editorial Office would like to apologize for any inconvenience caused to the readers by these changes. The changes do not affect the scientific results. The published version will be updated on the article webpage, with a reference to this Correction.

## Figures and Tables

**Figure 1:**
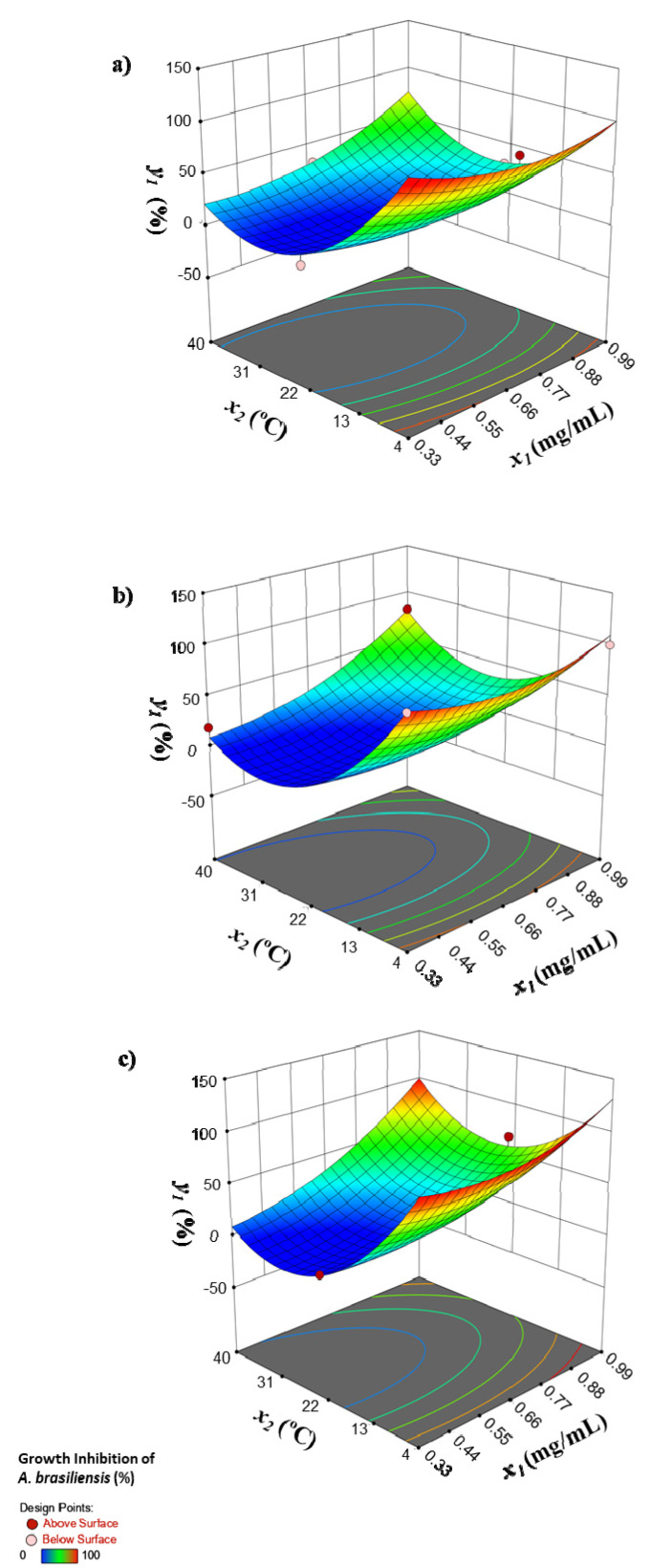
Growth inhibition of *A. brasiliensis* (%) as a function of the concentration of the biosurfactant (*x_1_*) and temperature (*x_2_*) of incubation for different incubation times (*x_3_*): (**a**) 5, (**b**) 8 and (**c**) 11 days.

**Table 2:** Operational conditions used in this study, expressed as coded dimensionless and uncoded independent variables: concentration of the biosurfactant (*x*_1_), temperature (*x*_2_), and incubation time (*x*_3_); the results obtained for the dependent variables: *y*_1_ (% of growth inhibition of *A. brasiliensis*) and *y*_2_ (% of growth inhibition of *C. albicans*). (−1, minimum value of the variable within the range; 0, central value of the variable within the range; 1, maximum value of the variable within the range).

	Coded Independent Variable			Uncoded Independent Variable			Dependent Variable	
Exp	*x_1_*	*x_2_*	*x_3_*	*x_1_* (mg/mL)	*x_2_* (°C)	*x_3_* (Days)	*y_1_*	*y_2_*
1	0	−1	−1	0.66	4	5	100.00	0.00
2	0	1	−1	0.66	40	5	31.71	62.55
3	0	−1	1	0.66	4	11	100.00	0.00
4	0	1	1	0.66	40	11	26.91	49.95
5	−1	−1	0	0.33	4	8	100.00	17.79
6	−1	1	0	0.33	40	8	18.88	40.89
7	1	−1	0	0.99	4	8	100.00	0.00
8	1	1	0	0.99	40	8	82.52	76.33
9	−1	0	−1	0.33	22	5	0.00	6.42
10	−1	0	1	0.33	22	11	0.00	0.00
11	1	0	−1	0.99	22	5	30.43	11.07
12	1	0	1	0.99	22	11	67.87	0.00
13	0	0	0	0.66	22	8	0.00	0.00
14	0	0	0	0.66	22	8	0.00	0.00
15	0	0	0	0.66	22	8	0.00	0.00

**Table 4:** Fungicidal and fungistatic conditions of the biosurfactant extracted from the CSW against *A. brasiliensis* and *C. albicans* in refrigerator storage (4 °C) and room temperature (25 °C) (* fungistatic effect, ** fungicidal effect).

	*A. brasiliensis*			*C. albicans*		
T (°C)	t (Days)	Biosurfactant Concentration (mg/mL)	Growth Inhibition (%)	t (Days)	Biosurfactant Concentration (mg/mL)	Growth Inhibition (%)
4	5.0	0.33	100 ^**^	5	0.99	17.9
4	10.0	0.35	100 ^**^			
25	10.2	0.99	50 ^*^	5	0.99	20.0
25	11.0	0.99	57 ^*^			
